# Case Report: Massive Hemoptysis From a Spontaneously Regression Inflammatory Bronchial Polyp

**DOI:** 10.3389/fmed.2022.875311

**Published:** 2022-05-31

**Authors:** Yuichiro Iwamoto, Haruka Takenouchi, Katsumasa Koyama, Ryo Shirai, Hideaki Kaneto, Koichi Tomoda

**Affiliations:** ^1^Department of Diabetes, Endocrinology and Metabolism, Kawasaki Medical School, Kurashiki, Japan; ^2^Department of General Internal Medicine 1, Kawasaki Medical School, Okayama, Japan

**Keywords:** bronchial inflammatory polyp, lung hemorrhage, massive hemoptysis, spontaneous disappearance, case report

## Abstract

**Background:**

Bronchial inflammatory polyps are usually treated by surgical operation or with steroids and/or antibiotics, and it is quite rare that such polys spontaneously disappear without any treatment. This report shows a rare case with a bronchial inflammatory polyp which caused massive hemoptysis but spontaneously disappeared without any treatment.

**Case Presentation:**

A 66-year-old man with type 2 diabetes mellitus and a history of cough and asthma suddenly developed massive hemoptysis while smoking and was brought to an emergency room in our institution. In bronchoscopy on admission, a polypoidal elevated lesion was observed in the left upper lobe bifurcation. Pulsatile hemorrhage from a polypoidal elevated lesion was observed upon stimulation of passage of the bronchoscope. Bronchoscopy performed 25 days after discharge showed no evidence of active bleeding and a tendency toward reduction of the elevated lesion. In bronchoscopy performed 106 days after the initial hospitalization, the bronchial inflammatory polyp completely disappeared.

**Conclusions:**

We should bear in mind the possibility of spontaneous disappearance of bronchial inflammatory polyps causing some serious symptoms such as massive hemoptysis and repeated bloody sputum. Finally, we should select the best therapy for bronchial inflammatory polys based on each patient's background and conditions in clinical practice.

## Introduction

Bronchial inflammatory polyps are non-neoplastic elevated lesions composed of inflammatory granulation tissue and are considered to be a rare disease. Causes of bronchial inflammatory polyps include infection, local chronic inflammation, chronic irritation by airflow, allergic reaction of the airway mucosa, airway foreign body, and idiopathic with no apparent cause. Bronchial inflammatory polyps are often accompanied by local hemorrhage and characterized by symptoms such as bloody sputum, hemoptysis and cough. Since they are usually found as hemorrhagic masses, it is very important to differentiate them from primary bronchial tumors in clinical practice. In general, bronchial inflammatory polyps are treated by surgical operation or with steroids and/or antibiotics, and it is quite rare that such polys spontaneously disappear without any treatment. This report shows a rare bronchial inflammatory polyp which caused massive hemoptysis but spontaneously disappeared without any treatment in a subject with type 2 diabetes mellitus.

## Case Report

**Figure 1:**
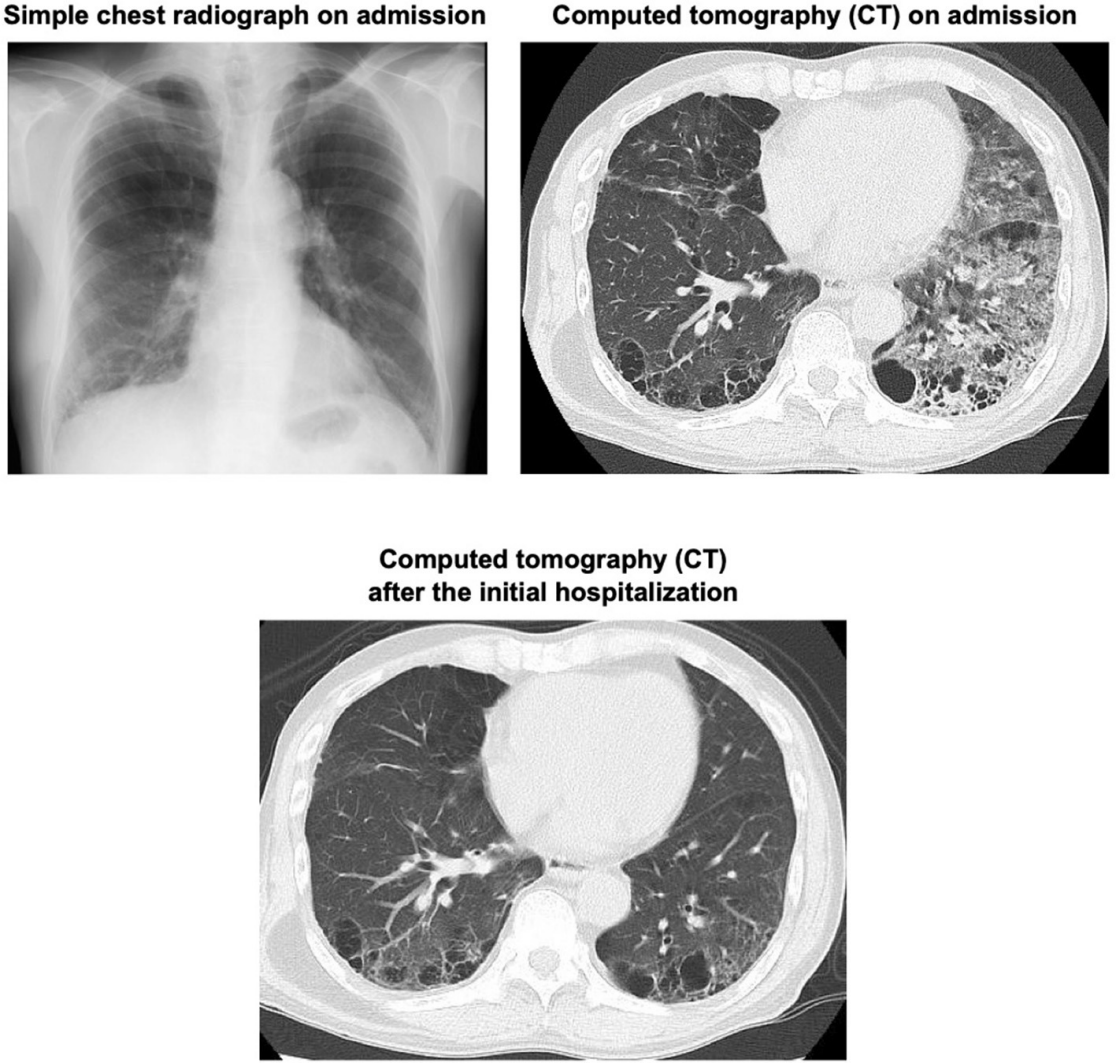
A simple chest photograph at the time of emergency transport showed reticular shadows in the bilateral lower lung fields (upper left panel). Computed tomography (CT) at the time of emergency transport showed an infiltrative shadow with air bronchograms in the right lower lobe. There were emphysematous changes in the bilateral lungs (lower right panel). CT performed 106 days after initial discharge showed that the infiltrative shadow in the right lower lobe had disappeared (lower panel).

**Figure 2:**
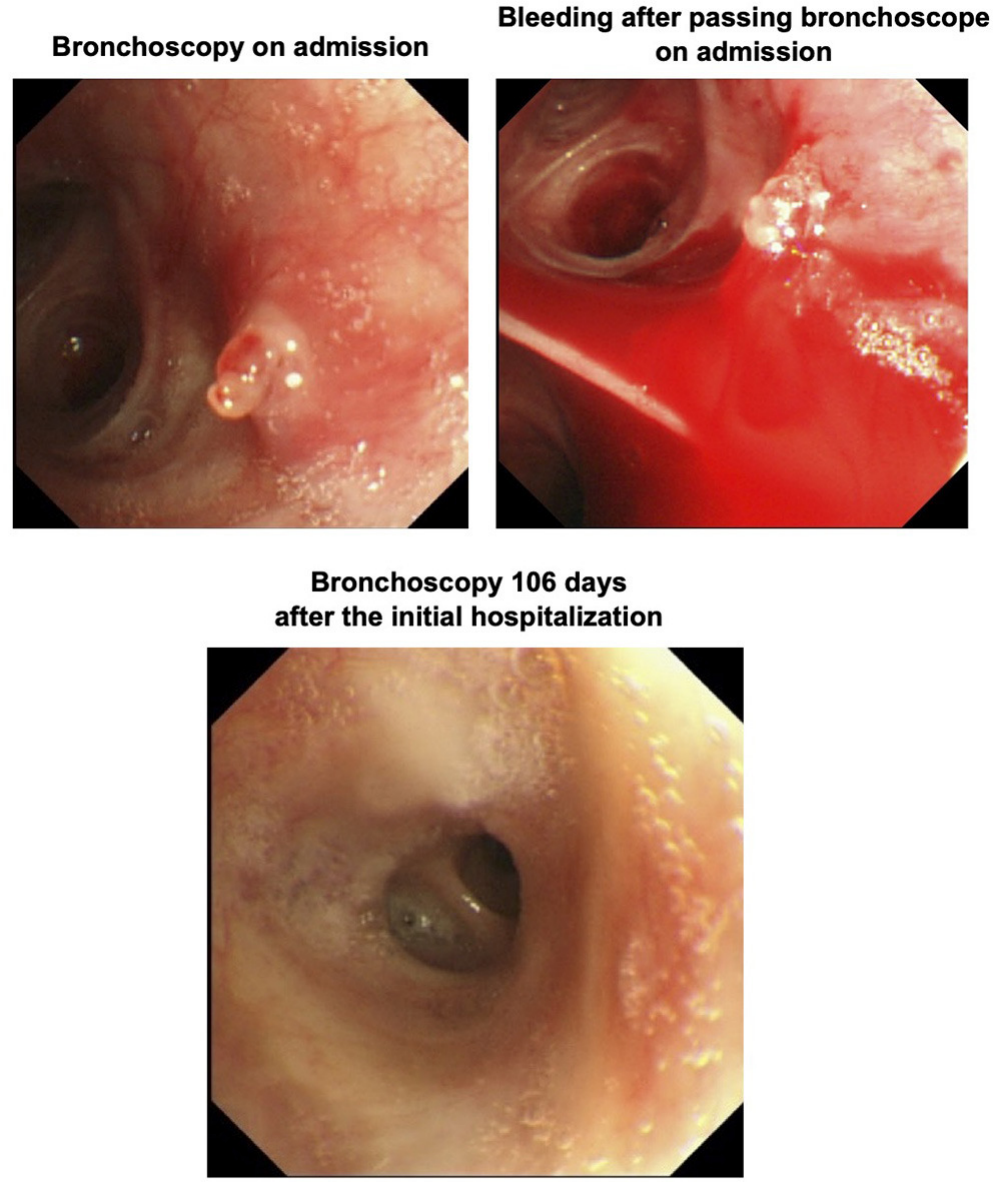
Bronchoscopy performed on the day of admission showed a polypoid elevated lesion at the bifurcation of the left upper lobe (upper left panel). Passage of bronchoscope revealed pulsatile bleeding around the polypoid lesion (upper right panel). Bronchoscopy performed 106 days after the initial hospitalization revealed that the elevated lesion completely disappeared (lower panel).

A 66-year-old man with type 2 diabetes mellitus and a history of cough and asthma suddenly developed massive hemoptysis of more than 1L while smoking 2 days before. After then, he was brought to an emergency room in our institution because of repeated bloody sputum. His smoking history was 60 pack years. At the time of admission, the patient was taking 50 mg of vildagliptin for diabetes mellitus. His height and body weight were 168 cm and 52.9 kg, respectively. His blood pressure, pulse rate, and respiratory rate were 120/50 mmHg, 97 beats per minute, and 18 beats per minute, respectively. In chest auscultation, there were no fine or coarse crackles, and there were no other physical findings of note. The findings of blood tests at the time of emergency transport are shown in Table 1. His hemoglobin level was 15.7 g/dL and there was no anemia. His coagulation functions are all in the normal range. Liver and renal functions were almost within the normal range. Serum C-reactive protein (CRP) was mildly elevated to 0.40 mg/dL. Diabetes markers were as follows: blood glucose level, 112 mg/dL; HbA1c, 7.5%. Sputum cultures did not reveal any pathologic micro-organisms. A chest radiograph on admission demonstrated reticular shadows in the right lower lung fields. Sputum cultures did not reveal any pathologic micro-organisms. A chest radiograph on admission demonstrated reticular shadows in the right lower lung fields (Figure 1, upper left panel). Chest computed tomography (CT) on admission showed frosted shadows in the dorsal left upper lobe and left lower lobe. The left lower lobe showed ground-glass opacity and fibrotic and cystic changes (Figure 1, upper right panel). In contrast-enhanced CT, however, there were no abnormal vessels or neoplastic lesions in the bronchial wall. To examine the cause of massive hemoptysis and subsequent repeated bloody sputum, bronchoscopy was performed on the day of admission. As the results, a polypoidal elevated lesion was observed in the left upper lobe bifurcation (Figure 2, upper left panel). There were no exposed blood vessels in the tracheal lumen, but passage of the bronchoscope revealed pulsatile bleeding from the periportal area of the polyp (Figure 2, upper right panel). Thrombin was applied under bronchoscopy and hemostasis was achieved. To minimize recurrence of bleeding from mechanical irritation and cough, the patient was empirically treated with anti-tussive and anti-secretory agents such as carbazochrome (90 mg/day), carbocisteine (1500 mg/day), cloperlastine (60 mg/day) with symptomatic relief. There was no sign of infection, and considering the risk of hyperglycemia, we decided not to administer antimicrobial agents and steroids. The patient was discharged from the hospital on the 7th day after admission because hemoptysis was not observed at all after the start of medication.

**Table 1:** Laboratory data on admission.

**Variable**	**Result**	**Reference range**	**Variable**	**Result**	**Reference range**
**Blood biochemistry**			**Peripheral blood**		
Total protein (g/dL)	6.8	6.6–8.1	White blood cells (/μL)	6940	3300–8600
Albumin (g/dL)	4.1	4.1–5.1	Neutrophil (%)	67.5	28.0–78.0
Globulin (g/dL)	2.7	2.2–3.4	Red blood cells (×10^4^/μL)	512	386–492
Total bilirubin (mg/dL)	0.5	0.4–1.5	Hemoglobin (g/dL)	15.7	11.6–14.8
AST (U/L)	12	13–30	Hematocrit (%)	46.3	35.1–44.4
ALT (U/L)	9	7–23	Platelets (×10^4^/μL)	20.9	15.8–34.8
LDH (U/L)	244	124–222	**Infectious marker**		
ALP (U/L)	332	106–322	CRP (mg/dL)	0.26	<0.14
γ-GTP (U/L)	23	9–32	QFT	(–)	
BUN (mg/dL)	11	8–20	**Diabetes marker**		
Creatinine (mg/dL)	0.79	0.46–0.79	Plasma glucose (mg/dL)	112	
Cholinesterase (U/L)	207	201–421	Hemoglobin A1c (%)	7.5	4.9–6.0
Uric acid (mg/dL)	4.5	2.6–5.5	**Urinary test**		
Sodium (mmol/L)	141	138–145	Urinary pH	6.5	5.0–7.5
Potassium (mmol/L)	3.6	3.6–4.8	Urinary protein	(±)	-
Chloride (mmol/L)	104	101–108	Urinary sugar	(–)	-
**Coagulation system test**			Urinary ketone body	(2+)	-
PT (sec)	11.6	9.3–12.5	Urinary bilirubin	(–)	-
PT-INR	0.97	0.85–1.13	Urinary blood	(–)	-
APTT (sec)	28.5	26.9–38.1			
Fibrinogen	281	160–380			

*AST, aspartate aminotransferase; ALT, alanine aminotransferase; LDH, lactate dehydrogenase; ALP, alkaline phosphatase; γ-GTP, γ-glutamyl transpeptidase; BUN, blood urea nitrogen; PT, prothrombin time; APTT, activated partial thromboplastin time; QFT, QuantiFeron test; CRP, C-reactive protein*.

**Figure 3:**
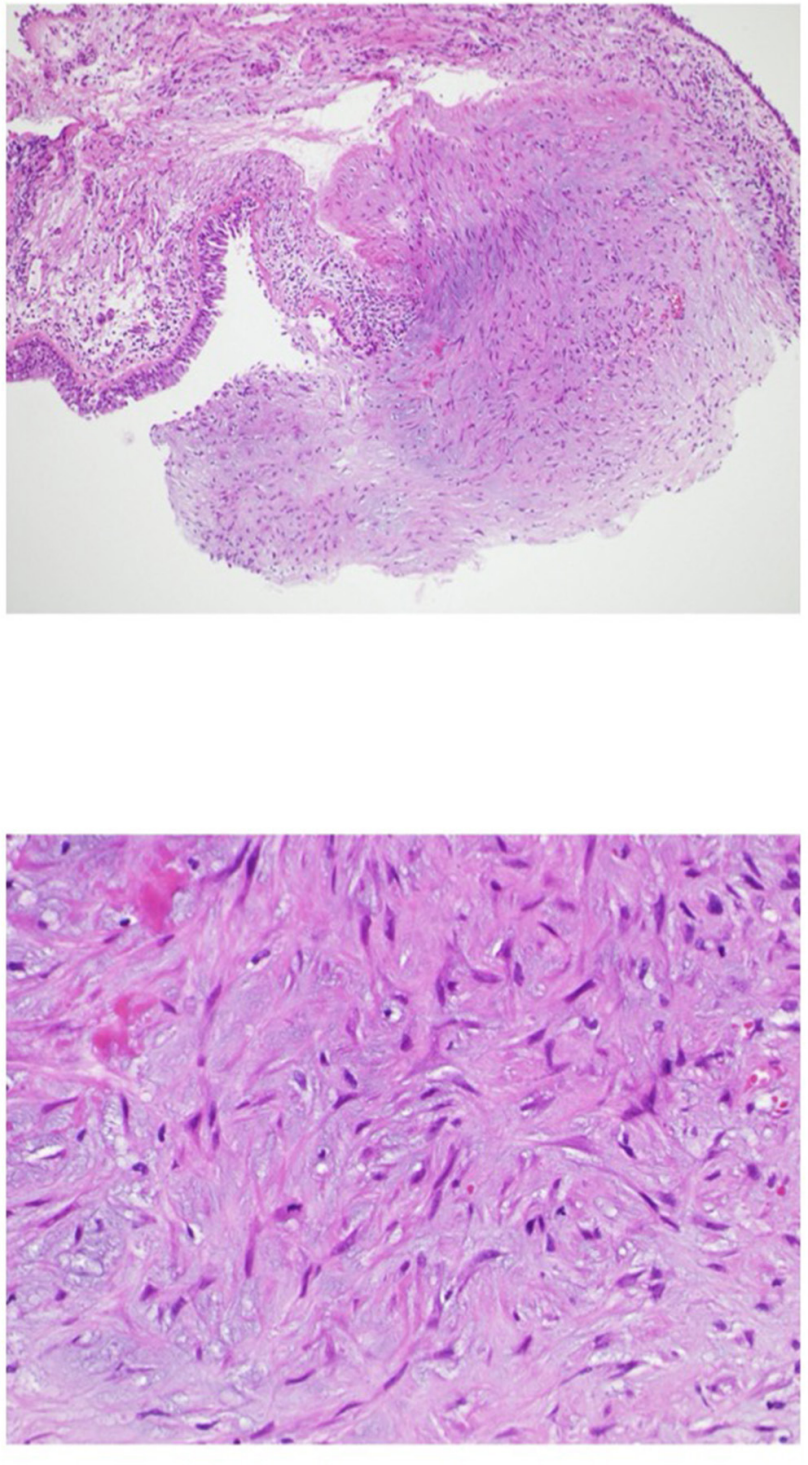
Findings in biopsy specimen from the hemorrhagic polypoid lesion. The upper panel shows a weakly magnified image of HE staining, and the lower panel shows a strongly magnified image of HE staining. Foci of reactive myofibroblast proliferation were observed from the bronchial mucosa to the bronchial lumen.

The patient underwent a follow-up bronchoscopy on day 25 post discharge which showed no evidence of active bleeding. The polypoidal nodule appeared less pronounce and an endobronchial biopsy was performed. Histopathology demonstrated a foci of reactive myofibroblast growth from the bronchial mucosa to the endobronchial palace suggestive of a bronchial inflammatory polyp (Figure 3). The patient was instructed to quit smoking and after then he did not experience hemoptysis. To examine the alteration of the lesion, bronchoscopy was performed 106 days after the initial hospitalization. As the results, the bronchial inflammatory polyp completely disappeared although this polyp previously caused massive hemoptysis and repeated bloody sputum in this subject (Figure 2, lower panel). On follow-up chest imaging, the lower lobe infiltrates had resolved with residual underlying fibrotic changes that are currently being evaluated for suspected smoking-related interstitial lung disease (Figure 1, lower panel).

## Discussion

Bronchial inflammatory polyps were first reported in 1929 as a pathological diagnosis in bronchoscopic biopsies and were defined pathologically as being completely covered by normal bronchial mucosal epithelium and continuous epithelium, with submucosal connective tissue interstitium (1, 2). Causes of bronchial inflammatory polyps include airway irritation due to infection, chronic irritation by airflow, allergic reaction in the bronchial mucosa, direct mechanical irritation, and idiopathic cases in which the cause cannot be identified (3–6). In this case, the patient had a history of cough and asthma, and it seemed that repeated hemoptysis due to smoking brought about airway allergy and airway irritation by airflow.

Although neoplastic lesions such as malignant disease, bronchial tuberculosis, and bronchial papilloma can be listed as differential diseases for bronchial inflammatory polyps, it is difficult to diagnose bronchial inflammatory polyps based on bronchoscopic findings alone, and histopathological diagnosis by biopsy is ultimately necessary (7). It has been reported that about 8% of bronchial inflammatory polyps disappear spontaneously, while polyps themselves may cause bloody sputum, hemoptysis and cough, and thus are usually treated with some method. Treatment methods include conservative treatment, surgical resection, and endoscopic treatment such as forceps removal under a bronchoscope and cauterization with Nd-YAG laser (6, 7). In rare cases, major bleeding may occur, and it has been reported that in some cases inflammatory edema may disappear spontaneously with antibacterial agents and steroids (8). However, administration of steroids to patients with concomitant diabetes mellitus may pose a risk of secondary infection due to hyperglycemia. Furthermore, this case report clearly indicates the possibility that inflammatory polys spontaneously disappear without any surgical operation or treatment with steroids and/or antibiotics.

Taken together, although bronchial inflammatory polyps with heavy hemoptysis are usually treated by surgical operation or steroid therapy, both surgical operation and steroid therapy can induce several complications or side effects. Especially in subjects with poorly controlled diabetes mellitus, it would be better to avoid surgical operation or usage of steroid, because it is well known that both surgical operation and steroid therapy often aggravate glycemic control. Therefore, as observed in the present case, we should bear in mind the possibility of spontaneous disappearance of bronchial inflammatory polyps bringing about some serious symptoms such as massive hemoptysis and repeated bloody sputum. Finally, we should select the best therapy for bronchial inflammatory polys based on each patient's background and conditions in clinical practice.

## Data Availability Statement

The original contributions presented in the study are included in the article/supplementary material, further inquiries can be directed to the corresponding author/s.

## Author Contributions

YI wrote the paper. HT, KK, RS, HK, and KT were involved in teaching and revising the paper content. All authors contributed to the article and approved the submitted version.

## Conflict of Interest

The authors declare that the research was conducted in the absence of any commercial or financial relationships that could be construed as a potential conflict of interest.

## Publisher's Note

All claims expressed in this article are solely those of the authors and do not necessarily represent those of their affiliated organizations, or those of the publisher, the editors and the reviewers. Any product that may be evaluated in this article, or claim that may be made by its manufacturer, is not guaranteed or endorsed by the publisher.
